# Ultrasound-guided Hernia Reduction—Preventing Surgery for an Incarcerated Ventral Hernia: A Case Report

**DOI:** 10.5811/cpcem.47051

**Published:** 2026-07-20

**Authors:** Arion Lochner, Elizabeth Stovicek, Nicholas Kman, Sarah Petelinsek, Jennifer Cotton

**Affiliations:** * University of Utah, Department of Emergency Medicine, Salt Lake City, Utah; † Case Western Reserve University, Department of Emergency Medicine, Cleveland, Ohio; ‡ Ohio State University, Department of Emergency Medicine, Columbus, Ohio; § University of Utah, Spencer Fox Eccles School of Medicine, Salt Lake City, Utah

**Keywords:** point-of-care ultrasound, ultrasound guidance, hernia management, hernia reduction, case report

## Abstract

**Introduction:**

Hernias are a common presenting complaint in the emergency department. If they are not promptly identified and reduced, patients may need urgent surgery, which entails an increase in morbidity and mortality. The diagnosis and reduction of hernias can be challenging due to a patient’s large body habitus. In this case, we demonstrate the utility of using point-of-care ultrasound (POCUS) to detect an abdominal wall defect and provide real-time guidance to reduce a hernia and avoid surgery in a patient who was a poor surgical candidate.

**Case Report:**

A 48-year-old morbidly obese male with multiple comorbidities presented with abdominal pain at the location of a known ventral hernia. Computed tomography (CT) of the abdomen confirmed a ventral hernia; however, three different physicians attempted reduction and were unsuccessful. We then used POCUS to identify the ventral hernia and the abdominal wall defect and to guide the subsequent reduction efforts. Guidance of the reduction started with locating the abdominal wall defect, which allowed for gentle, steady pressure to be applied at the site of the defect and visualization of the successful reduction in real time. This led to immediate improvement in the patient’s symptoms.

**Conclusion:**

The success of this reduction highlights how POCUS can be used to improve the success rate for manual ventral hernia reduction without the need for confirmatory CT. It can also help to avoid increased morbidity and mortality from unplanned, unoptimized surgical hernia repair in high-risk patients.

## INTRODUCTION

Hernias are a common presenting complaint in the emergency department (ED), with an estimated lifetime incidence of nearly 10%.^1^ The initial diagnosis can be made on physical examination; however, physical exam may be limited by body habitus, which can reduce sensitivity for detecting hernias. The sensitivity of physical exam remains suboptimal even in non-obese patients.^2^ Point-of-care ultrasound (POCUS) has been shown to be more sensitive than physical exam for diagnosing hernias at the bedside.^3^,^4^ In inguinal hernias, for example, POCUS has a sensitivity of 96.6%, specificity of 84.4%, and positive predictive value of 92.6%.^8^–^11^ Given its accuracy and ability to be quickly performed at the bedside, POCUS adds significant value to patient care by facilitating earlier diagnosis during the initial evaluation.

While accurate diagnosis of hernias is critical, timely reduction is even more crucial. Reducible hernias can be manually reduced while incarcerated hernias cannot. However, this distinction is based on physical exam and manual technique rather than imaging characteristics. As a result, a hernia may be reducible for one clinician and incarcerated for another. If a hernia cannot be reduced, urgent surgical intervention is necessary to prevent progression to bowel ischemia and strangulation. Once bowel ischemia and strangulation develop, surgery becomes emergent to prevent the progression to bowel perforation and sepsis.^1^ Incidence of mortality for strangulated hernias is 1.4–13.4%.^5^ Given that 15% of abdominal wall hernias alone require emergent inpatient repair, these nonreducible hernias cause significant morbidity and mortality.^7^ Preventing the progression from incarceration to strangulation and reducing the number of emergent hernia repairs could substantially improve patient outcomes.^5^

Ultrasound offers utility beyond making the initial diagnosis of a hernia. Point-of-care ultrasound can be a helpful adjunct for challenging manual hernia reductions. And it can be used to identify the abdominal wall defect, including characterizing its location and size as well as delineating the boundaries of the herniated bowel—especially in cases where the hernia is difficult to palpate. Another benefit of POCUS is that it allows for continuous, real-time visualization of the hernia during reduction attempts. This allows clinicians to directly observe changes in hernia size and confirm successful reduction as the bowel slides back through the defect. Another key advantage of POCUS is its rapid availability, allowing it to be incorporated into the initial bedside exam. This can lead to earlier diagnosis and treatment without the delays associated with obtaining computed tomography (CT) imaging, thereby reducing time to intervention and the risk of progression to strangulation. Additionally, color-flow Doppler can be used to assess bowel perfusion before attempting reduction, helping to identify signs of strangulation.^6^

Building upon prior knowledge of ultrasound for the diagnosis of hernias, we used the above methods to quickly reduce an incarcerated ventral hernia that could not be reduced manually by multiple physicians. The case we present here illustrates a novel technique for ultrasound-guided ventral hernia reduction. This has the potential to help prevent progression to strangulation, decrease the need for emergent surgical repairs, and lessen the associated morbidity and mortality from emergent hernia repairs.

## CASE REPORT

A 48-year-old morbidly obese male (body mass index > 40 kg/m^2^ (reference range, 8.5–24.9 kg/m^2^) presented with multiple comorbidities including poorly controlled diabetes with hemoglobin A1c of 12.9% (< 5.7%), seizures, immune thrombocytopenic purpura status postsplenectomy, kidney transplant in 1990, avascular necrosis of femur head, cystectomy with ileal conduit, nicotine dependence, and a previous ventral hernia repair.

*CPC-EM Capsule*

What do we already know about this clinical entity?

*Point-of-care ultrasound (POCUS) is more sensitive for diagnosing hernias at bedside than physical exam.*

What makes this presentation of disease reportable?

*This case demonstrates POCUS as a mechanism to reduce a previously “incarcerated” hernia.*

What is the major learning point?

*POCUS may be a useful tool to reduce hernias without surgical intervention.*

How might this improve emergency medicine practice?

*This report highlights how ultrasound can be used to improve the success rate for manual ventral hernia reduction without the need for confirmatory computed tomography.*

The patient presented complaining of being unable to reduce a known ventral hernia that was typically reducible. He complained of two weeks of lower abdominal pain and nausea but had no complaints of vomiting or fever. He had a bowel movement the day before and was passing flatus. On exam he was not tachycardic, tachypneic, hypotensive, or febrile. He had normal bowel sounds and a mildly tender abdomen, and the ventral hernia was believed to be palpated to the left of a midline scar. Laboratory studies revealed a blood glucose of 251 mg/dL (reference range, 70–99 mg/dL) and a mild leukocytosis but were otherwise within normal limits. Computed tomography of the abdomen and pelvis confirmed a small ventral hernia.

Three separate physicians—including emergency and surgical attendings—attempted blind manual reduction of the hernia. During each attempt, steady pressure was applied for several minutes with the patient positioned in steep Trendelenburg position. Intravenous (IV) pain and antiemetic medications were also administered. However, the patient’s body habitus made it difficult to clearly palpate the ventral hernia and abdominal wall defect, complicating the reduction efforts. Ultimately, the hernia could not be reduced using traditional manual techniques at the bedside.

**Image 1:**
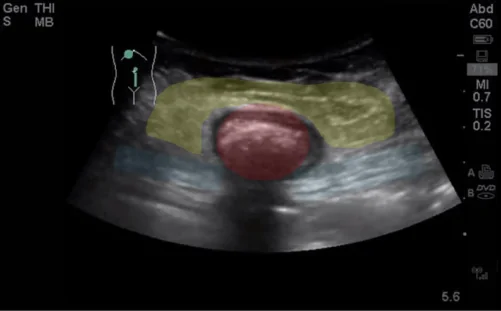
Ultrasound confirming failure of multiple attempts to manually reduce the hernia. A loop of bowel (red) is seen protruding from the abdominal muscle layer (blue) and into subcutaneous fat (yellow). A clear defect is seen in the abdominal musculature deep to the herniated bowel.

Given the limitations of the physical exam, the care team requested ultrasound assistance to localize the hernia and confirm the failure of prior reduction attempts. Ultrasound was first used to identify the ventral hernia and verify that previous efforts had been unsuccessful (Image 1). The abdominal wall defect was measured at 1.72 cm, and the hernia at its widest point measured approximately 4 cm.

**Image 2:**
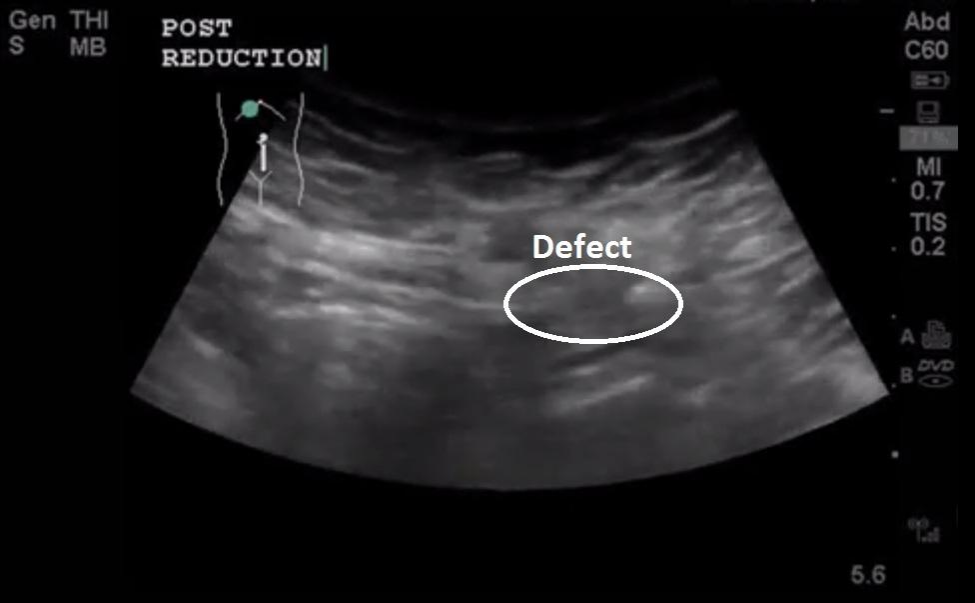
Following the use of ultrasound to identify the abdominal wall defect and gently apply pressure to the herniated bowel, the hernia reduction was confirmed using ultrasound. While the defect in the abdominal muscles can still be seen, it is now free of bowel.

Using a technique similar to that used for ultrasound-guided peripheral IV line placement, the operator aligned their fingers with the visualized defect and applied gentle, steady pressure directly over the site. Real-time visualization allowed for more precise targeting of the herniated bowel and helped distinguish it from surrounding tissue. Within one minute, the hernia was successfully reduced. The patient experienced immediate relief and reported a subjective sense that the hernia had been reduced. Follow-up ultrasound confirmed resolution of the hernia (Image 2), and CT ordered by the surgical team provided additional confirmation. Despite challenges related to body habitus, ultrasound guidance enabled a successful reduction that had previously not been possible using conventional methods.

The patient was admitted for observation and discharged by hospital day 2 after he tolerated full intake per os. Multiple consultants advised that he would likely have had a poor outcome from emergency surgery before medical optimization of his multiple comorbidities. Even after optimization he would still have been at high risk for poor wound healing and poor postoperative outcomes. A successful ultrasound-guided ventral hernia reduction spared this patient the need for a potentially dangerous surgery and for the year-long period during which the encounter was monitored.

## DISCUSSION

This case illustrates how ultrasound can be used as both a diagnostic and therapeutic tool for the care of ventral hernias. This novel approach allows physicians to both confirm manual reduction success or failure without waiting for CT and aid in the actual reduction for challenging hernias. Physicians can easily identify bowel that is difficult to palpate, locate the abdominal wall defect, and apply pressure directly over the defect and bowel to improve the effectiveness of reduction efforts. Direct visualization of the ventral hernia and abdominal wall defect also allows for real-time feedback on the effectiveness of the amount and direction of pressure being applied. Furthermore, ultrasound gives immediate feedback on reduction success. This could potentially lead to earlier reduction of ventral hernias through earlier diagnosis, easier assessment of reduction success or failure, and greater overall manual reduction rates following failed attempts at blind reduction.

This method of incorporating ultrasound into ventral and all hernia reductions could lead to better resource use for acute hernia care. Making the diagnosis and using ultrasound to aid the reduction of hernias at the bedside during an initial ED evaluation would reduce the need for CT to confirm the presence and reduction of hernias, especially those that are difficult to palpate. In addition, using ultrasound to guide hernia reduction may reduce the number of hernias that must urgently undergo surgical repair. This could lead to decreased use of resources and shortened length of stay. Additionally, this would decrease hospital costs associated with admission and emergency surgical repair. If earlier reduction of hernias further reduced the number of hernias that progress along the spectrum toward strangulation, this technique would also reduce the associated morbidity and mortality from strangulated hernias. The technique appears particularly valuable for those patients who may be poor surgical candidates, such as the one highlighted in our case, who are at high risk of developing complications. Overall, ultrasound guidance for manual hernia reduction has the potential to reduce healthcare costs and resource use.

In summary, this innovative approach uses ultrasound to first identify the failure of manual hernia reduction and then to guide the subsequent reduction efforts. Guidance of the reduction starts with locating the abdominal wall defect. Direct visualization of the defect allows for gentle, steady pressure to be applied at the site of the defect and visualize the successful reduction in real time at the bedside without the need for confirmatory CT. The success of this method significantly improved the outcome for our patient who was at high risk for complications from an unplanned, unoptimized surgical hernia repair.

## CONCLUSION

The success of the use of point-of-care ultrasound as described in this report highlights how ultrasound can be used to improve the success rate for manual ventral hernia reduction without the need for confirmatory CT. It can also help to avoid increased morbidity and mortality from unplanned, unoptimized surgical hernia repair in high-risk patients.
